# Severe Hepatic Injury Due to Dengue Fever Without Warning Signs in a Patient With Intellectual Disability: A Case Report

**DOI:** 10.7759/cureus.90778

**Published:** 2025-08-22

**Authors:** Khalil F Miyajan, Fahad S Alsallum, Mohammed S Alhuthali, Ahlam A Alsomali

**Affiliations:** 1 Department of Internal Medicine, King Faisal Specialist Hospital and Research Centre, Jeddah, SAU; 2 Department of Internal Medicine/Geriatric Medicine/Hospice and Palliative Medicine, King Faisal Specialist Hospital and Research Centre, Jeddah, SAU

**Keywords:** acute liver failure, aedes, arboviral disease, dengue fever (df), insect bite, liver injury, mosquito bite

## Abstract

Dengue fever is a mosquito-borne arboviral disease prevalent in tropical countries that presents as a spectrum of symptoms ranging from mild fever to severe life-threatening illness with multi-organ dysfunction. Hepatic involvement is common in dengue fever, but it is usually mild and self-limiting. However, in some cases, it can present with acute liver failure, leading to a diagnostic dilemma. We present the case of a young man with developmental delay and a history of mosquito bites, who presented with dengue fever and severe viral hepatitis. A 20-year-old Saudi man presented to the emergency department with elevated liver enzyme levels, fever, decreased oral intake, nausea, abdominal pain, dysuria, dizziness, and confusion. He had been bitten by a mosquito 10 days before symptom onset. The patient was initially treated conservatively with paracetamol and hydration, but he developed progressive liver dysfunction. Laboratory evaluation revealed elevated liver enzyme levels, thrombocytopenia, and hyperferritinemia, with positive NS1 suggestive of acute liver injury. However, the viral hepatitis panel and infection workup gave negative results, and abdominal ultrasonography showed no evidence of biliary obstruction or structural liver disease. In conclusion, dengue fever is a prevalent tropical disease that can cause severe hepatic injury and acute liver failure. Clinicians should be aware of the possibility of severe liver injury in patients with dengue fever, particularly in those with risk factors for severe disease. Prompt recognition and appropriate management of these conditions are essential for preventing fatal outcomes.

## Introduction

Dengue fever is a febrile illness caused by infection with one of four dengue virus serotypes, which are transmitted by Aedes mosquitoes. Dengue virus serotype 2 is associated with the most severe disease [1]. It is an arboviral disease with a substantial burden in tropical countries and a rising prevalence: in 1997 and 2008, approximately 75 million and 150 million dengue infections, respectively, were reported globally [2]. This increase is a result of poor sanitation, insufficient health systems, and increased international travel, all of which have aided Aedes mosquito production. Asia accounts for 70% of the cases, with India alone accounting for 34% of the global total [2]. In Saudi Arabia, dengue fever is endemic in the western region, particularly in Jeddah and Makkah, with a seroprevalence of around 30.8% and an incidence estimated at 11 cases per 100,000 population in 2022 [3]. 

Dengue fever is diagnosed based on fever and at least two of the following symptoms: ocular pain; headache; muscle, joint, or rash pain; and leukopenia. The incubation period is three to seven days, and the symptoms are described as three phases: febrile (two to seven days), critical (three to seven days of sickness with vascular leakage, shock, and/or organ damage), and convalescent [4]. The laboratory diagnosis of dengue fever is established by detection of dengue virus non-structural protein 1 (NS1) antigen, or by identification of dengue virus RNA through reverse transcriptase-polymerase chain reaction during the acute febrile phase; alternatively, a positive dengue virus-specific immunoglobulin M and/or a rising titer of immunoglobulin G antibodies on serology can confirm the diagnosis in the appropriate clinical context [5].

The liver is the organ most frequently affected by the disease [4]. Dengue affects the liver through several mechanisms, including direct viral invasion of hepatocytes and Kupffer cells, an exaggerated immune response driven by a T cell-mediated cytokine storm, and impaired hepatic perfusion due to circulatory failure [6]. The extent of liver damage varies from mild transaminitis without symptoms to acute liver failure (ALF). Co-infection of dengue fever with viral hepatitis (hepatitis A virus (HAV) or hepatitis B virus (HBV)) has also been reported, leading to more severe hepatic dysfunction, higher transaminase elevations, jaundice, and an increased risk of acute liver failure [7-8].

In this report, we discuss the case of a young man with developmental delay who presented with dengue fever and severe viral hepatitis in the absence of warning signs.

## Case presentation

A 20-year-old Saudi man living in Jeddah and working in a fast-food restaurant presented to the Emergency Department (ED) with a history of elevated liver enzyme levels after being referred from the family medicine clinic on December 6, 2022. The patient was in his usual state of health until five days prior to presentation, when he complained of objective fever reaching 40.2℃ that was partially relieved with analgesia and associated with decreased oral intake, nausea, abdominal pain, dysuria, and one episode of dizziness and confusion, as reported by the family. The patient reported being bitten by a mosquito in his work area 10 days ago. He had a history of epilepsy well-controlled with levetiracetam and intellectual disability with an ankyrin-3 gene mutation. He was taking levetiracetam at 750 mg twice daily; his dose was last adjusted in September 2021, when it was increased from 500 mg to the current dose. The patient denied any history of weight loss, loss of appetite, jaundice, or bleeding from any orifices. Furthermore, the patient denied any history of drinking unclean water or establishing contact with a sick patient.

Three days after symptom onset, the patient visited a family clinic and underwent routine investigations; his laboratory tests showed elevated liver enzyme levels (Table 1). He was treated conservatively with paracetamol and hydration and was advised to follow up with the family clinic after one day. On the follow-up visit, symptoms were seen to be resolved; however, when the hepatic profile was examined again, he showed a progressive increase in liver enzyme levels. Therefore, the patient and his family were advised to visit the ED for further workup regarding the elevated liver enzyme levels.

**Table 1:** Laboratory parameter trend at different points of time FMC: Family Medicine Clinic; ALT: Alanine transaminase; AST: Aspartate transaminase; ALP: Alkaline phosphatase; N/A: Not available.

Test	Reference Range	FMC 1st Visit (04/12)	FMC 2nd Visit (05/12)	Admission Day 1 (06/12)	Admission Day 2 (07/12)	Post-discharge One-Week Follow-up	Post-discharge Three-Week Follow-up
ALT/AST (U/L)	ALT: 7-56, AST: 10-40	164/158	1054/1213	1398/1511	1612/1503	143/40	32/37
ALP (U/L)	40-129	76	95	95	123	108	106
Total Bilirubin (μmol/L)	3-20	5	8	13	12	8	7
Platelet (×10^9^/L)	150-450	94	77	131	126	N/A	202
Hemoglobin (g/L)	130-170	160	170	171	161	N/A	156
Leukocytes (×10^9^/L)	4.0-10.0	2.02	3.55	5.98	6.84	N/A	6.55
Creatinine (μmol/L)	60-120	109	92	74	88	N/A	N/A
Ferritin (ug/L)	30-400	N/A	N/A	N/A	15987	549	204

At presentation at the ED, the patient was alert, oriented to time and place, and not in pain or distress. He did not appear jaundiced, and there were no visible petechiae, bruises, or ecchymosis on his body. His body temperature was 36.8℃, blood pressure, 102/67 mmHg, and pulse was 70 beats per minute. Moreover, the oxygen saturation was 100% in room air, and the respiratory rate was 20 breaths per minute. Abdominal examination revealed mild tenderness in the upper left quadrant. Organomegaly and masses were absent. The oral examination revealed no evidence of gum bleeding or ulcers. Chest, cardiovascular, and neurological examinations were unremarkable.

Laboratory evaluation

Laboratory evaluations revealed normal leukocytes (Table 1). However, peripheral blood morphology showed reactive lymphocytes with 10% abnormal cells, a platelet count of 131 10^9^/L, aspartate transaminase (AST) of 1511 U/L, alanine transaminase (ALT) of 1398 U/L, albumin of 44 g/dL, alkaline phosphatase of 95 U/L, total bilirubin of 8 μmol/L, and international normalized ratio (INR) of 0.9. The acetaminophen (APAP) level was less than 5 mg/dL. The NS1 test was positive, and the viral hepatitis panel and broad infectious workup gave negative results. Anti-nuclear antibody, anti-smooth muscle antibody, anti-mitochondrial antibody, liver function, kidney function, and microsomal antibody tests also gave negative results. The levels of ferritin and C-reactive protein were 15,987 u/L and 8 mg/L (normal, 0-5), respectively. Serum creatinine and hemoglobin levels were within the normal ranges at the time of admission. Abdominal ultrasonography showed a normal liver and spleen with no focal lesions and a normally distended gallbladder with a normal-sized common bile duct

Treatment and follow-up

The patient was treated conservatively with hydration and discharged on the second day, and at the one-week follow-up with Gastroenterology, the patient had no active complaints. Repeated laboratory investigations showed significant improvements in his hepatic profile and ferritin levels (Table 1). Laboratory examination at the three-week follow-up showed normalization of his hepatic profile and ferritin levels (Table 1).

## Discussion

Dengue fever presents with varying degrees of severity. According to the 2009 World Health Organization (WHO) classification, dengue infection is categorized based on severity into three groups: dengue without warning signs, dengue with warning signs (including abdominal pain, persistent vomiting, fluid accumulation, mucosal bleeding, lethargy, liver involvement, and rising hematocrit with decreasing platelet count), and severe dengue, which involves severe plasma leakage, significant bleeding, or organ failure [9]. Classic dengue fever typically manifests with fever, intense headache, retro-orbital pain, extreme fatigue, severe muscle pain (myalgia), and joint pain (arthralgia) [10].

Diagnosing medical illnesses in individuals with developmental delays can be challenging for healthcare providers, as these individuals may have difficulty communicating their symptoms and underlying medical conditions that are not easily recognizable. For instance, due to our patient’s developmental delay, it was difficult to obtain a high-quality history to reach a diagnosis, and the history was mainly obtained from the caregiver.

An important possibility is levetiracetam-induced liver injury, which is dose-dependent and remarkably rare, with a likelihood score of C (probable cause). However, levetiracetam-induced liver injury occurs from one week to five months after starting the medication [6], and our patient had been using this medicine since 2019, the last dose change being in 2021. The other important differential diagnosis was shock liver and/or compensated shock; however, the patient remained normotensive with a normal heart rate. Electronic medical records of the past two years revealed normal blood pressures.

Workplaces, such as fast food restaurants, may play a significant role in the spread of dengue fever [11]. This may be due to several factors, including poor hygiene and the presence of stagnant water in restaurants, which can serve as breeding grounds for mosquitoes, thereby increasing the chances of spread of the virus.

Patients with dengue typically present abdominal pain, nausea, emesis, anorexia, and hepatomegaly with or without jaundice, along with mild transaminitis, hyperbilirubinemia, hypoalbuminemia, and an increased INR. Our patient lacked these classic signs, and the severity of transaminitis made his presentation unique. Although elevated AST and ALT levels have been reported in 63%-97% and 45%-96% of patients with dengue, respectively, transaminitis occurs in only 4% of cases [3,12]. Transaminase values that are fivefold above the normal value are usually observed in hemorrhagic fever more often than in classic dengue [13,14], unlike in this case. Our patient's peak AST and ALT levels were 1503 and 1612 U/L, respectively, which raised our suspicion of viral hepatitis; however, the hepatitis workup returned negative results.

The patient's severe liver injury was likely multifactorial. In addition to the direct hepatotoxic effects of the dengue virus, hemodynamic instability was suspected as a contributing factor, given the patient's episode of dizziness and confusion, raising concerns about possible ischemic hepatitis. Impaired liver function can also reduce acetaminophen (APAP) metabolism, increasing the risk of APAP-related hepatotoxicity even with repeated small doses [15]. Initially treated for a viral infection, the patient received a total of 5.2 g of paracetamol to manage fever before concerns about APAP-related liver injury arose. Although the total dose was subsequently reduced to below 5 g, it may have played a role in hepatic injury. 

Dengue fever is managed supportively, as no specific antiviral therapy is available. Oral fluid intake is encouraged, while intravenous fluids and blood products are administered as needed. However, fluid management requires careful monitoring during the critical phase of illness, as vascular leakage combined with excessive fluid administration can lead to respiratory complications, as seen in this patient. N-acetylcysteine (NAC) is another therapeutic option that mitigates hepatic damage by scavenging free radicals, improving systemic hemodynamics, and enhancing tissue oxygen delivery [16]. However, NAC administration was not deemed necessary in this case.

## Conclusions

Dengue virus infection leads to a mild-to-moderate elevation of liver transaminases in almost all cases. However, this case clearly demonstrates that dengue fever can cause acute hepatitis without plasma leakage or overt bleeding and could be a cause of extremely severe hepatic transaminitis. Acute liver failure can be fatal in most cases; however, in this case, the patient was managed with supportive care and had a complete recovery of liver function with no residual symptoms.
